# Pathogen analysis of pertussis-like syndrome in children

**DOI:** 10.1186/s12879-020-05074-8

**Published:** 2020-05-19

**Authors:** Wenjing Gu, Kun Wang, Xinxing Zhang, Chuangli Hao, Yanhong Lu, Min Wu, Sainan Chen, Yanyu He, Jun Xu, Xuejun Shao, Yuqing Wang

**Affiliations:** 1 Department of Respiration, Children’s Hospital of Soochow University, No. 303 Jing De Road, Suzhou, 215003 China; 2 Department of Clinical laboratory, Children’s Hospital of Soochow University, Suzhou, 215003 China

**Keywords:** *B. Pertussis*, *Mycoplasma pneumoniae*, Pathogen, Pertussis-like syndrome

## Abstract

**Background:**

The aim of the study was to identify the pathogens, in addition to bordetella pertussis (*B. pertussis*), which cause pertussis-like syndrome in children and to compare clinical presentation between those with *B. pertussis* and pertussis-like syndrome.

**Methods:**

A cross-sectional analysis was conducted from March 2016 to September 2018. In total, 281 children with suspected pertussis infections were enrolled in this study. Multi-pathogen detection was performed.

**Results:**

In total, 281 children were enrolled including 139 males and 142 females. Among them, 149 (53.0%) were *B. pertussis* positive, and 72 (15.6%) children tested positive for other pathogens. *Mycoplasma pneumoniae* (MP, 27 cases) was the most common causative pathogen in pertussis-like syndrome, followed by human rhinovirus (HRV, 23 cases), *Streptococcus pneumoniae* (SP, 13 cases), *Haemophilus influenzae* (HI, 12 cases) and parainfluenza virus 3 (Pinf-3, 9 cases). Children in the *B. pertussis* group had a higher rate of vaccination and longer hospital stay (*P* < 0.05). *B. pertussis* was more likely to be detected in winter than other pathogens, but this difference was not significant (*P* = 0.074). The number of white blood cells, neutrophils and blood platelets was significantly higher in children in the *B. pertussis* than in the pertussis-like group (*P* < 0.05). In addition, the percentage of CD3-CD19+ cells was significantly higher in the *B. pertussis* group (*P* = 0.018).

**Conclusion:**

About half of the children with pertussis-like syndrome were *B. pertussis* positive. MP was the second most common causative pathogen followed by HRV, SP, HI and Pinf-3. Children infected with *B. pertussis* had longer hospital stay and higher numbers of white blood cells, neutrophil and blood platelets compared with other pathogens.

## Background

Pertussis is one of the top ten fatal infections in children, causing serious and potentially fatal complications, especially in very young infants [1, 2]. It is a respiratory disease caused by infection with *Bordetella pertussis (B. pertussis)*. The well-known symptoms of pertussis include repeated paroxysmal cough, inspiratory whoop, and post-cough vomiting [3]. However, these clinical symptoms are not limited to patients with *B. pertussis* infection. Other pathogens such as adenovirus (ADV), influenza virus (IV), and *Mycoplasma pneumoniae* (MP) also can cause similar clinical symptoms [4], collectively known as pertussis-like syndrome.

Pertussis-like syndrome can occur at all ages but is more common in children. It can be very unpleasant for patients, especially young infants and their parents, as symptoms frequently interfere with daily activities and cause significant sleep disturbance. Especially in the paroxysmal stage characterized by spasmodic cough followed by post-tussive whooping and vomiting, the effect of available medications is poor leading to anxiety in parents. It is difficult to distinguish the symptoms of infection with *B. pertussis* from infection with viruses. In addition, there is a lack of information around the etiology of pertussis-like syndrome worldwide. As such, a greater understanding of the pathogens that cause pertussis-like syndrome is important to inform treatment decision making.

In this study, we aimed to identify the causative pathogens associated with pertussis-like syndrome and to compare clinical presentation between those with *B. pertussis* and pertussis-like syndrome in children admitted to the Children’s hospital of Soochow university.

## Methods

### Study design and population

This was a cross-sectional study designed to identify the causative pathogens associated with pertussis-like syndrome. Children with suspected pertussis who were admitted to the Children’s Hospital of Soochow University from March 2016 to September 2018 were enrolled in this study. The clinical criteria for suspected pertussis are cough lasting for > 2 weeks with one or more of the following symptoms: whoop and staccato cough, apneic paroxysm or post-tussive vomiting. The exclusion criteria were historical diagnosis of chronic lung disease, congenital heart disease, immunodeficiency or preterm birth at ≤34 weeks’ gestation. In addition, 91 children admitted to the childcare unit for health examination were enrolled in the control group, including 75 boys and 16 girls. The average age was (0.67 ± 0.58) years old. Routine blood tests and cellular immunity results were collected.

### Sample collection

Nasopharyngeal aspirates were obtained from each patient within 18 h after admission using a sterile plastic catheter, which was briefly inserted into the lower pharynx via the nasal cavity. These samples were used for detection of common microorganisms, such as respiratory syncytial virus (RSV), ADV, influenza viruses A and B (IV-A and B), parainfluenza viruses 1, 2, and 3 (Pinf-1, 2, 3), human metapneumovirus (hMPV), human bocavirus (HBoV), human rhinovirus (HRV), MP, and bacteria.

Blood samples were collected immediately after admission for routine tests (Sysmex XS-500i, Hua Sin Science Co., Ltd., Guangzhou, China), liver and kidney function (ADVIA 2400, Siemens Healthineers, America), and cellular immunity.

### B. Pertussis detection

*B. pertussis* DNA was detected in nasopharyngeal aspirates by real-time polymerase chain reaction (PCR). Pathogen detection was achieved using a TaqMan genomic assay and fluorescent real-time PCR (BIO-RAD iCycler, USA). *B. pertussis* DNA was determined using two PCR assays, each specific for an independent region of the *B. pertussis* genome: (i) the insertion sequence IS481 and (ii) the pertussis toxin S1 (ptxA) promoter region. IS481 is a very sensitive target for screening *B. pertussis* because it is the most common insertion sequence, with multiple copies per genome [5, 6]. PtxA is highly specific but less sensitive because it usually exists as a single or occasionally double copy [7, 8]. Oligonucleotide primers targeting the ptxA promoter of *B. pertussis* (PTp1 and PTp2, Table 1) were used to amplify a 191-bp product, while primers targeting the insertion sequence IS481 of B. pertussis (BP-1 and BP-2MOD, Table 1) were used to amplify a 145-bp product. All primers and probes were purchased from Sangon Biotech (Shanghai) Co., Ltd. (Shanghai, China). Positive, negative and water blank controls were set for each PCR amplification. The judgement of positive results is generally < 34 cycles. Higher than this number, but with an amplification curve, and the curve is smooth, we would repeat the experiment. If the results of the two experiments were the same, a positive result was recorded, otherwise negative. B. pertussis infection was defined as IS481 PCR positive, whether ptxA PCR is positive or not.

**Table 1:** Primers and probes used for duplex real-time PCR

Target gene	Primer	Sequence (5′ to 3′)
ptxA-Pr	PTp1	CCAACGCGCATGCGTGCAGATTCGTC
ptxA-Pr	PTp2	CCCTCTGCGTTTTGATGGTGCCTATTTTA
IS481	BP-1	GATTCAATAGGTTGTATGCATGGTT
IS481	BP-2MOD	TGGACCATTTCGAGTCGACG

### Other microbiological analyses

Ten viruses and bacteria (including *MP)* were also tested in nasopharyngeal aspirates. Bacteria were tested by inoculating nasopharyngeal aspirates on blood plates that were read after incubating for 18–20 h. Bacterial growth was > 10^3^ colony forming units/ml was considered significant. Viruses including RSV, ADV, IV-A and B and Pinf-1, 2, 3 were investigated by immunofluorescence tests using D^3^ Ultra™ Respiratory Virus Screening and LD Kit (Diagnostic Hybrids, Ohio, USA). A positive result was defined as over five inclusion bodies analyzed under a fluorescence microscope. MP and the various viruses were examined by PCR (Nucleic acid amplification fluorescent reagent kit, Ann Gene Co., Guangdong, China) according to the manufacturer’s instructions.

### Cellular immunity

Venous blood was anticoagulated by heparin and antibodies were added to whole blood (anti-CD3+ and anti-CD19+ labeled with cyanobacteria-5 antibody, anti-CD4+, anti-CD23+ and anti-CD25+ labeled with fluorescein isothiocyanate, and anti-CD8+ antibody labeled with algal red protein). After mixing, the cells were incubated in a dark chamber and lysed with hemolysin, followed by further incubation in a dark chamber. Flow cytometry was performed using a BECKMAN COULTER flow cytometer and a specific kit (Immunotech, France).

### Clinical data collection

Detailed demographic and clinical data such as age, gender, pertussis vaccination status, previous antibiotic consumption, family history of pertussis, and clinical symptoms were obtained from questionnaires that were answered by patients’ parents and from medical records.

### Statistical analysis

Enumeration data are expressed as mean ± standard deviation (SD). Measurement data are expressed as a percentage (%). Statistical analysis was performed using SPSS 18.0 software (SPSS Inc., Chicago, IL); the rank sum test was used for comparison among groups and one-way analysis of variance was used for comparison. *P* < 0.05 was considered statistically significant.

## Results

### Study population

During the study period, a total of 281 children presenting with pertussis-like syndrome were included in the study. Among them, 139 were male and 142 were female. The male to female ratio was approximate 1:1.02. Age ranged from 1 month to 12 years old with a median age of 5 months (interquartile rane: 3–8.5 months). From the 281 nasopharyngeal aspirates, 149 (53.0%) were *B. pertussis* positive (140 cases for both IS481 and PtxA positive and 9 cases for only IS481 positive), referred to as the *B. pertussis* group. Another 132 children who were infected with pathogens other than *B. pertussis* or who tested negative overall were classified as the pertussis-like group.

### Comparison between pertussis and pertussis-like syndrome

#### Clinical manifestation

Children in the pertussis-like group were younger than those in the *B. pertussis* group, but there was no significant difference between them (*P* = 0.291). The pertussis-like group seemed to include more girls while the *B. pertussis* group included more boys, but with no significant difference (*P* = 0.056). Children in the *B. pertussis* group had a higher rate of vaccination compared with the pertussis-like group (43.2% vs 60.4%, *P* = 0.004) (this does not include children under 3 months old who do not need to be vaccinated). Children who were not vaccinated against pertussis or who were not fully vaccinated according to age were judged to not have received a scheduled vaccination. There was no significant difference in the scheduled vaccination rate between the two groups (66.3% vs 78.4%, *P* = 0.072). Children in the *B. pertussis* group had longer hospital stay compared with the pertussis-like group (9.91 ± 3.74 days vs 11.37 ± 4.90, *P* = 0.006). *B. pertussis* was more likely to be detected in winter than other pathogens, but there was no significant difference in seasonality between the two groups (*P* = 0.074). There were no significant difference in clinical manifestation between the two groups with regards to symptoms such as paroxysmal cough, post-cough vomiting, inspiratory whoop and so on. Pertussis encephalopathy and pulmonary hypertension were not identified in any children. Data are presented in Table 2.

**Table 2:** Difference in clinical manifestations between pertussis and pertussis - like syndrome

	pertussis-like group *n* = 132	*B. pertussis* group *n* = 149	*Χ*^*2*^/*t*	*P*
age	0.68 ± 1.39	0.84 ± 1.15	−1.058	0.291
Sex M/F	57/75	82/67	3.933	0.056
vaccination	57 (43.2%)	90 (60.4%)	8.321	0.004
Scheduled vaccination rates	55 (66.3%, total 83)	87 (78.4%, total 111)	3.552	0.072
hospital stay (d)	9.91 ± 3.74	11.37 ± 4.90	−2.778	0.006
Admission Season				
Spring	52 (39.4%)	58 (38.9%)	6.941	0.074
Summer	59 (44.7%)	56 (37.6%)		
Autumn	15 (11.4%)	15 (10.1%)		
Winter	6 (4.5%)	20 (13.4%)		
Clinical criteria				
paroxysmal cough	117 (88.6%)	133 (89.3%)	0.028	1.000
apnea	1 (0.8%)	0 (0%)	1.133	0.470
inspiratory whoop	20 (15.2%)	16 (10.7%)	1.220	0.288
asphyxia	1 (0.8%)	0 (0%)	1.133	0.470
turn red in the face	109 (82.6%)	118 (79.2%)	0.515	0.545
Severe pneumonia	3 (2.3%)	3 (2.0%)	0.023	1.000
post-cough vomiting	32 (24.2%)	37 (24.8%)	0.013	1.000
respiratory failure	1 (0.8%)	2 (1.3%)	0.227	1.000
Paroxysmal cyanosis	23 (17.4%)	29 (19.5%)	0.193	0.759
pertussis encephalopathy	0 (0%)	0 (0%)	–	–
hyoxemia	5 (3.8%)	6 (4.0%)	0.011	1.000
pulmonary arterial hypertension	0 (0%)	0 (0%)	–	–

#### Laboratory examination

The number of white blood cells and neutrophils was significantly higher in children in the *B. pertussis* group and the pertussis-like group than the control group (*P* < 0.001). This phenomenon was more obvious in the *B. pertussis* group than the pertussis-like group (15.94 ± 9.40 vs 19.06 ± 9.99, *P* = 0.008; 9.34 ± 6.46 vs 11.52 ± 7.73, *P* = 0.011). Children infected with *B. pertussis* had a significantly higher number of blood platelets than patients in the pertussis-like group (455.05 ± 122.68 vs 487.01 ± 133.38, *P* = 0.038). Both groups had a significantly higher number of blood platelets than patients in the control group (*P* < 0.001). There were no significant differences in the number of lymphocytes or liver function as measured by serum creatine kinase of MB type (CKMB) or C-reactive protein (CRP). For cellular immunity, the percentage of CD3+ cells was significantly lower in the *B. pertussis* group than the control group (63.40 ± 9.34 vs 66.89 ± 6.58, *P* = 0.001) but no significant difference was identified compared with the pertussis-like group (*P* = 0.157). The percentage of CD3-CD19+ cells was significantly higher in the *B. pertussis* group than the other two groups (*P* = 0.018 and *P* < 0.001). Both the pertussis-like group and the *B. pertussis* group had a significantly lower percentage of CD3 + CD8+ cells than the control group (*P* < 0.001). The percentage of CD3-(CD16 + CD56) + cells was slightly lower than in the *B. pertussis* group, but with no significant difference (*P* = 0.152). Data are presented in Table 3.

**Table 3:** Difference in laboratory examination among pertussis, pertussis - like syndrome and control group

	pertussis-like group n = 132	*B. pertussis* group n = 149	Control group *n* = 91	*Χ*^*2*^/*F*/*t*	*P*
white blood cell	15.94 ± 9.40^a,b^	19.06 ± 9.99^b^	8.44 ± 1.88	44.640	< 0.001
neutrophil	9.34 ± 6.46^a,b^	11.52 ± 7.73^b^	2.14 ± 1.00	65.687	< 0.001
lymphocyte	5.27 ± 4.14	6.09 ± 5.20	5.43 ± 1.44	1.516	0.221
blood platelet	455.05 ± 122.68^a,b^	487.01 ± 133.38^b^	327.87 ± 81.56	53.170	< 0.001
elevated ALT	38 (28.8%)	30 (20.1%)	–	2.857	0.096
elevated AST	23 (17.4%)	24 (16.1%)	–	0.087	0.873
elevated CRP	8 (6.1%)	5 (3.4%)	–	1.161	0.395
elevated CKMB	65 (49.2%)	74 (49.7%)	–	0.033	0.905
CD19 + CD23+	13.82 ± 6.04	13.62 ± 5.78	–	0.281	0.779
CD3+	64.97 ± 8.83	63.40 ± 9.34^b^	66.89 ± 6.58	4.711	0.010
CD3 + CD4+	40.24 ± 8.68^b^	38.98 ± 9.63	37.95 ± 6.78	1.892	0.152
CD3 + CD8+	21.68 ± 5.82^b^	21.54 ± 6.70^b^	24.87 ± 4.68	10.331	< 0.001
CD3-(CD16 + CD56)+	8.56 ± 5.74	7.37 ± 5.36	8.29 ± 4.44	1.895	0.152
CD3-CD19+	24.58 ± 9.87^a^	27.34 ± 9.35^b^	23.27 ± 6.00	6.736	0.001
CD4/CD8	2.03 ± 0.82^b^	2.01 ± 0.83^b^	1.58 ± 0.43	11.701	< 0.001

^a^compared with B. pertussis group, *P* < 0.05; ^b^ compared with control group, *P* < 0.05

#### Chest radiograph and pulmonary auscultation

The most common abnormal chest radiological findings in both groups were bilateral diffuse interstitial infiltrations and patchy shadows with no significant differences between the two groups. Moist rales and wheezing were most common in children with *B. pertussis* infection. Data were presented in Table 4.

**Table 4:** Chest radiograph and pulmonary auscultation comparison between pertussis and pertussis - like syndrome

	pertussis-like group n = 132	*B. pertussis* group n = 149	*Χ*^*2*^	*P*
Chest radiograph				
Patchy shadows	90 (68.2%)	102 (68.5%)	0.002	1.000
diffuse interstitial infiltrations	42 (31.8%)	47 (31.5%)		
*pulmonary auscultation*				
moist rales	41 (31.1%)	41 (27.5%)	13.706	0.003
wheezing	11 (8.3%)	12 (8.1%)		
moist rales+wheezing	8 (6.1%)	32 (21.5%)		
none	71 (53.8%)	64 (43.0%)		

#### Pathogens in children with pertussis-like syndrome

Among 132 children who had pertussis-like syndrome, 72 children tested positive for the pathogen (54.4%). Among them, 46 (63.9%) patients had simple infections and 26 (36.1%) patients had co-infections. MP was the most common pathogen in patients with pertussis-like syndrome. The most common virus was HRV, followed by Pinf-3, RSV, hBoV. The most common causative bacteria was *Streptococcus pneumoniae (SP)*, followed by *Haemophilus influenzae (HI).* The distribution of all pathogens is shown in Table 5.

**Table 5:** Pathogens detected in children with pertussis-like syndrome

Pathogen	Cases
Simple infection	46
*Mycoplasma Pneumonia*	13
Human Rhinovirus	9
Parainfluenza Virus 3	4
Respiratory Syncytial Virus	2
Human Bocavirus	2
Human Metapneumovirus	1
*Streptococcus pneumoniae*	7
*Haemophilus influenzae*	3
*Acinetobacter Baumannii*	1
*Staphylococcus aureus*	1
*Escherichia coli*	1
*Klebsiella Pneumoniae*	1
*Pseudomonas Aeruginosa*	1
Co-infection	26
Rhinovirus+*Streptococcus Pneumoniae*	4
Rhinovirus+*Haemophilus Influenzae*	3
*Mycoplasma Pneumonia*+*Haemophilus Influenzae*	3
*Mycoplasma Pneumonia*+Human Bocavirus	2
*Mycoplasma Pneumonia*+Rhinovirus	2
*Mycoplasma Pneumonia*+Parainfluenza Virus 3	1
*Mycoplasma Pneumonia*+*Moraxella Catarrhalis*	1
*Mycoplasma Pneumonia*+*Pseudomonas Aeruginosa*	1
Respiratory Syncytial Virus+Rhinovirus	1
Human Bocavirus+*Haemophilus Influenzae*	1
Parainfluenza Virus 3 + Rhinovirus+*Haemophilus Influenzae*	1
Parainfluenza Virus 3 + Rhinovirus+*Streptococcus Pneumoniae*	1
*Mycoplasma Pneumonia*+Parainfluenza Virus 3 + *Haemophilus Influenzae*	1
*Mycoplasma Pneumonia*+Human Bocavirus+*Streptococcus Pneumoniae*	1
*Mycoplasma Pneumonia*+Parainfluenza Virus 3 + Rhinovirus	1
Rhinovirus+*Staphylococcus Aureus*	1
*Mycoplasma Pneumonia*+ Rhinovirus+ *Staphylococcus Aureus* + *Escherichia Coli*+ *Haemophilus Influenzae*	1

#### Comparison between pathogen-positive and negative pertussis-like syndrome

There were no significant differences in clinical manifestations or laboratory results between patients with pathogen-positive and negative pertussis-like syndrome. There were also no significant differences with regards to age, sex ratio, vaccination history or hospital stay between the two groups. Data are presented in Table 6.

**Table 6:** Difference in clinical manifestations and laboratory examination between pathogen positive and negative pertussis - like syndrome among those who were B. pertussis negative

	Pathogen positive *n* = 72	Pathogen negative *n* = 60	*Χ*^*2*^/*t*	P
age	0.67 ± 1.48	0.69 ± 1.28	−0.064	0.949
Sex M/F	26/46	31/29	3.228	0.080
vaccination	33(45.8%)	24(40%)	0.454	0.597
hospital stay (d)	10.14 ± 3.54	9.63 ± 3.99	0.771	0.442
paroxysmal cough	66(91.6%)	51(85%)	1.444	0.277
apnea	0(0%)	1(1.7%)	1.209	0.455
inspiratory whoop	12(16.7%)	8(13.3%)	0.283	0.634
asphyxia	0(0%)	1(1.7%)	1.209	0.455
turn red in the face	61(84.7%)	48(80%)	0.507	0.498
Severe pneumonia	2(2.8%)	1(1.7%)	0.182	1.000
post-cough vomiting	18(25.0%)	14(23.3%)	0.050	0.842
respiratory failure	1(1.4%)	0(0%)	0.840	1.000
Paroxysmal cyanosis	12(16.7%)	11(18.3%)	0.063	0.822
pertussis encephalopathy	0(0%)	0(0%)	–	–
hyoxemia	3(4.2%)	2(3.3%)	0.062	1.000
pulmonary arterial hypertension	0(0%)	0(0%)	–	–
white blood cell	16.40 ± 10.71	15.39 ± 7.58	0.615	0.540
neutrophil	10.06 ± 7.05	8.47 ± 5.61	1.418	0.159
lymphocyte	4.95 ± 3.95	5.64 ± 4.37	−0.955	0.342
blood platelet	467.56 ± 128.98	440.05 ± 113.91	1.286	0.201
elevated ALT	19(26.4%)	19(31.7%)	0.445	0.565
elevated AST	11(15.3%)	12(20.0%)	0.507	0.498
elevated CRP	5(6.9%)	3(5.0%)	0.217	0.728
elevated CKMB	39(54.2%)	26(43.3%)	1.537	0.227
CD19 + CD23+	14.24 ± 5.98	13.31 ± 6.13	0.865	0.389
CD3	64.70 ± 9.02	65.29 ± 8.65	−0.370	0.712
CD3 + CD4+	40.13 ± 7.99	40.37 ± 9.52	−0.147	0.884
CD3 + CD8+	21.63 ± 5.76	21.74 ± 5.94	−0.095	0.925
CD3-(CD16 + CD56)+	8.40 ± 5.07	8.75 ± 6.59	−0.348	0.729
CD3-CD19+	25.17 ± 9.93	23.87 ± 9.84	0.735	0.464
CD4/CD8	1.99 ± 0.67	2.08 ± 0.98	−0.573	0.567

## Discussion

Pertussis is a series of respiratory symptoms caused by infection with *B. pertussis*, including paroxysmal fits of coughing, vomiting after coughing and deep inspirium. The intermittent symptoms of coughing can last for more than 28 days and up to 3 months [9]. However, in clinical practice, we found that other pathogens can also cause pertussis-like cough as part of pertussis-like syndrome. Stephanie Saiki-Macedo found that in Peru, there was a high prevalence of ADV, *B. pertussis,* MP and IV-B infection in patients with a probable diagnosis of pertussis [10]. In our study, detailed etiological examination was performed in 281 children who had typical pertussis-like syndrome. About half of the children were *B. pertussis* positive, and about a quarter of the children had infections with other pathogens. No pathogens were detected in around a quarter of the children tested. Our study found that, in addition to *B. pertussis*, the most common causative pathogen in children with pertussis-like syndrome children was MP, which was detected in more than one third of children. In addition, the rate of HRV, SP, HI and Pinf-3 infections was also high in pertussis-like syndrome.

Amal Al Maani compared pertussis and pertussis-like illness in Oman and found there was no significant difference in clinical criteria between them [11]. In our study, no children had pertussis encephalopathy or pulmonary arterial hypertension and there was no significant difference in other clinical manifestations between the pertussis-like group and the *B. pertussis* group. Spastic cough was more common in spring and summer and nearly half of these children had B. *pertussis.* However, more than 75% of children had B. *pertussis* infection in the winter. Therefore, the occurrence of whooping cough in winter should raise suspicion of *B. pertussis* infection.

Previous studies showed that leukocytosis, especially lymphocytosis was associated with mortalities and morbidities in patients with pertussis infection [12, 13]. In our study, the absolute lymphocyte count in the *B. pertussis* group was higher than the pertussis-like group, but with no significant difference. However, the absolute neutrophil count and blood platelet count in the B. *pertussis* group was significantly higher than in the pertussis-like group and control group. This may be associated with the time of onset. Joshua C. Eby studied the relationship between pertussis bacillus and neutrophils and found that the recruitment of neutrophils is suppressed by the pertussis toxin early in the course of the disease, but after the first week, the combined activities of pertussis toxin, lipooligosaccharide and adenylate cyclase toxin result in production of cytokines that generate an IL-17 response, promoting neutrophil recruitment [14]. In our study, most children with pertussis had been ill for more than a week before being hospitalized. This could explain the significant increase of neutrophils in the children with *B. pertussis* infection in our study. As for the relationship between pertussis and platelets, the study results are controversial. Pertussis toxin shows distinct early signalling events in platelet-activating factors leading to thrombocytosis and platelet activation through interaction with platelet glycoprotein Ib [15, 16]. However, other studies showed that platelet aggregation is suppressed by the *B. pertussis* adenylate cyclase toxin through increased intracellular cyclic adenosine monophosphate (cAMP), and this dysfunction resulted in hemorrhage [17]. In our study, children infected with *B. pertussis* had a significantly higher number of blood platelets, which may be related to the overactive immune inflammatory response following *B. pertussis* infection.

MP is one of the most common causes of community-acquired pneumonia in children. In older children, MP infection can cause lobular pneumonia with clinical manifestations including high fever and cough. However, in young children, chest radiographs often showed interstitial inflammation which mainly manifested as cough and wheezing. Many studies showed that *MP* can cause persistent cough [18, 19]. Wang K found that MP is an important infection in children with persistent cough but the course is shorter than pertussis [20], which is similar to our results. As both *B. pertussis* and MP are mainly treated with macrolide drugs like erythromycin or azithromycin, children with persistent cough or spasmodic cough can be treated empirically with these drugs in clinical practice.

The current study has some limitations. First, the sample size is still small. In the next stage, we will continue to enroll more children with pertussis-like syndrome and follow up them to explore the long-term prognosis of these children. Second, there are still limitations in pathogen detection. Our test did not include all the pathogen that can cause pertussis-like syndrome, like *Bordetella parapertussis*, which accounts for 10–14.9% of *Bordetella* [21]. We will detect more kinds of pathogens including *Bordetella parapertussis* later*.*

## Conclusion

This was a cross-sectional study designed to identify the etiologic agents of pertussis-like syndrome. About half of the children were *B. pertussis* positive. *MP* was the second most common among other identified pathogens, followed by HRV, SP, HI and Pinf-3. Children infected with *B. pertussis* had a longer hospital stay and higher numbers of white blood cell, neutrophils and blood platelets.

## Data Availability

The datasets used and/or analysed during the current study available from the corresponding author on reasonable request.

## Abbreviations

ADV: Adenovirus
HI: *Haemophilus influenzae*
hMPV: human metapneumovirus
HRV: Human rhinovirus
IV: Influenza virus
*MP*: *Mycoplsma pneumonia*
Pinf-3: Parainfluenza Virus 3
RSV: Respiratory syncytial virus
SD: Standard deviation
SP: *Streptococcus pneumoniae*
